# Children and adolescents with VACTERL association: health-related quality of life and psychological well-being in children and adolescents and their parents

**DOI:** 10.1007/s11136-019-02364-w

**Published:** 2019-11-18

**Authors:** A-M. Kassa, M. Dellenmark-Blom, J. Thorsell Cederberg, G. Engvall, H. Engstrand Lilja

**Affiliations:** 1 Department of Women’s and Children’s Health, Uppsala University, Uppsala, Sweden; 2 Department of Paediatric Surgery, University Children’s Hospital, Uppsala, Sweden; 3 Department of Pediatrics, Institute of Clinical Sciences, Sahlgrenska Academy, University of Gothenburg, Gothenburg, Sweden; 4 Department of Paediatric Surgery, The Queen Silvia Children’s Hospital SU/Östra, Gothenburg, Sweden; 5 Department of Clinical Neuroscience, Karolinska Institutet, Stockholm, Sweden; 6 The Multidisciplinary Pain Centre and Rehabilitation Medicine, Uppsala University Hospital, Uppsala, Sweden

**Keywords:** Congenital malformations, VACTERL association, Health-related quality of life, Psychological well-being, Children and adolescents, Parents

## Abstract

**Purpose:**

VACTERL association is a rare and complex condition of congenital malformations, often requiring repeated surgery and entailing various physical sequelae. Due to scarcity of knowledge, the study aim was to investigate self-reported health-related quality of life (HRQoL), anxiety, depression and self-concept in children and adolescents with VACTERL association and self-reported anxiety and depression in their parents.

**Methods:**

Patients aged 8–17 years with VACTERL association and their parents were recruited from three of four Swedish paediatric surgical centres during 2015–2019. The well-established validated questionnaires DISABKIDS, Beck Youth Inventories, Beck Anxiety Inventory and Beck Depression Inventory were sent to the families. Data were analysed using descriptives, *t* tests and multivariable analysis. Results were compared with norm groups and reference samples.

**Results:**

The questionnaires were returned by 40 patients, 38 mothers and 33 fathers. The mean HRQoL was *M* = 80.4, comparable to children with asthma (*M* = 80.2) and diabetes (*M* = 79.5). Self-reported psychological well-being was comparable to the norm group of Swedish school children, and was significantly higher than a clinical sample. Factors negatively influencing children’s HRQoL and psychological well-being were identified. The parents’ self-reports of anxiety and depression were comparable to non-clinical samples.

**Conclusions:**

Although children and adolescents with VACTERL association reported similar HRQoL to those of European children with chronic conditions, their psychological well-being was comparable to Swedish school children in general. Nevertheless, some individuals among both children and parents were in need of extra support. This attained knowledge is valuable when counselling parents regarding the prognosis for children with VACTERL association.

## Introduction

VACTERL association is a chronic condition of congenital malformations that coexist in a single patient. The acronym stands for vertebral defects (V), anorectal malformations (A), cardiac defects (C), tracheoesophageal fistula (TE), renal anomalies (R) and limb abnormalities (L). At least three of these conditions need to be present for a VACTERL diagnosis [1]. The diagnosis is rare with birth prevalence estimated to be 6.25/100,000 in Europe [2]. Multiple surgeries are often required during childhood [1] and various physical sequelae may follow, such as scoliosis, bowel dysfunction, gastro-oesophageal reflux, dysphagia, airway morbidity, decreased cardiac, renal or limb function [1, 3, 4]. Chronic conditions seem to have a negative impact on psychosocial health, academic functioning and social competence in children [5–8]. Psychosocial well-being might be reduced in children with chronic conditions as they often experience repeated stays in hospital, painful medical treatment, disrupted school attendance, and limitations on social and physical activities [9]. Furthermore, health-related quality of life (HRQoL) including “physical, emotional, mental, social and behavioural components of well-being and function” [10] might be affected. Contradictory HRQoL outcomes have been described in children and adolescents with mostly single malformations [11–19]. Similarly, negative psychological impact has been reported in children, adolescents and young adults with congenital malformations [20–26], while other studies did not find corresponding results [27, 28]. Negative associations between anxiety and depression and HRQoL have been reported in adolescents with congenital heart disease (CHD) [29].

Parents of children with gastrointestinal, urological, neurological [30] and cardiac [31, 32] malformations may experience various psychological impacts, such as psychological distress [30], anxiety [30–32], depressive symptoms [31–33] and traumatic stress [30, 32, 34].

All things considered, present knowledge is scarce about HRQoL and the psychological well-being in children with VACTERL association as well as in their parents. However, such knowledge is needed in order to provide awareness of how, and to what extent, children, adolescents and their parents are affected by VACTERL association in daily life and need extra support. The study aim was to investigate the HRQoL, self-reported anxiety, depression and self-concept in children and adolescents with VACTERL association and self-reported anxiety and depression in their parents.

## Materials and methods

### Participants and procedures

Children and adolescents aged 8–17 years with VACTERL association, were recruited from three out of four Swedish tertiary paediatric surgical centres. During 2015–2019 they were invited together with their parents, to participate in a questionnaire study. Furthermore, three families were recruited by an invitation conveyed through a peer organisation for families with a child with VACTERL association. Questionnaires addressing children, mothers and fathers, respectively, were sent to all identified eligible 64 families together with an information letter for parents and age-adapted information for children and adolescents. The study was approved by the Regional Ethical Review Board in Uppsala.

### Measures

The DISABKIDS Chronic Generic Measure-long version (DCGM-37) was used to assess the HRQoL of children aged 8–17 years and a corresponding parents’ proxy version was applied for each mother and father. The questionnaires cover mental, social and physical domains of HRQoL. Results from a European field study sample including children with chronic conditions of asthma, arthritis, dermatitis, diabetes, cerebral palsy, cystic fibrosis, epilepsy and their parents were available as reference material [10, 35]. The DCGM-37 comprises 37 items with a 5-graded Likert scale transformed to numerical values 1–5, where higher values indicate better HRQoL. For the six subscales included we used positive names in line with suggestion from Chaplin et al. [36] namely: *independence, inner strength* (originally emotion), *social inclusion, equality* (originally social exclusion), *physical ability* (originally limitation) and *treatment* to make the results more comprehensible. The raw scores (RS) received from each subscale and the total summed up score were recoded to transformed raw scores (TRS) ranging from 0 to 100. Internal consistency has been reported as Cronbach’s alpha 0.70–0.87 in the six subscales and test–retest reliability has been found to be satisfactory [35].

Beck Youth Inventories (BYI) contain five subscales assessing thoughts, feelings and behaviours related to emotional and social impairment in children and adolescents aged 7–18 years. The inventories used in this study were Beck Youth Inventory-Anxiety (BYI-A), measuring worries about school, the future, reactions of others, fears and physiological symptoms related to anxiety, Beck Youth Inventory-Depression (BYI-D), referring to feelings of sadness and guilt, negative thoughts about self, life and the future, and sleeping difficulties, and Beck Youth Inventory-Self-concept (BYI-S), assessing perceived competence, potency, and positive self-worth [37]. Internal consistency for the inventories has been reported to be *α* = 0.89–0.94 and test–retest reliability has been found to be satisfactory. Each inventory consists of 20 items with a 4-graded Likert scale from 0 = “never” to 3 = “always”, reflecting level of agreement with the provided item statements during the two previous weeks. For the BYI-A and the BYI-D, high values indicate higher levels of anxiety and depression, respectively. For the BYI-S, a high value implies a more positive self-concept. Percentiles in BYI-A and BYI-D are interpreted in accordance with the manual, with < 74th percentile as average, 75th–89th as slightly elevated and ≥ 90th as very elevated anxiety and depression. For BYI-S, percentiles ≥ 90th are interpreted as high, 26th–89th as average, 11th–25th as somewhat low and ≤ 10th as very low self-concept. Reference values are provided for a norm group of Swedish schoolchildren (*n* = 2358) and a Swedish clinical sample of children with psychiatric diagnoses [38].

Beck Anxiety Inventory (BAI) [39] and Beck Depression Inventory (BDI-II) [40] were enclosed for each parent. Both instruments consist of 21 items with a 4-graded Likert scale from 0 = “never” to 3 = “always”. Internal consistency has been reported to be *α* = 0.88 for the BAI and *α* = 0.91 for the BDI-II in Norwegian non-clinical samples and temporal stability has been demonstrated for both scales [39, 40]. Guidelines for interpretation of anxiety levels suggest that scores between 0 and 7 indicate minimal anxiety, 8–15 mild anxiety, 16–25 moderate anxiety and 26–63 severe anxiety [40]. The corresponding levels for depression are 0–13 minimal depression, 14–19 mild depression, 20–28 moderate depression and 29–63 severe depression [40]. Reference values for BAI were found from Finnish [41], US [42] and Spanish non-clinical samples [43] and for BDI from a Norwegian non-clinical sample [40].

In addition to the questionnaires, a parent-reported form was attached with an open question concerning the child’s/adolescent’s physical complications or symptoms during the last year.

### Processing data

To verify data entries, the individual scores from the questionnaires were double-entered into two different spread sheets which were thereafter compared by using an excel function. In DCGM-37 one missing value is accepted in each subscale when obtaining the TRS according to the SPSS syntax provided. If double alternatives were marked in an item these values were excluded. Due to many missing values in the subscale of treatment in our study group, it was excluded and 31 items were used for the overall score of HRQoL. According to the instructions for BYI, missing values were accepted in two items per scale and replaced through imputation of the mean value of the existent responses [38]. The same principle was used for BAI and BDI. When double answers were marked in BYI the worst value was chosen according the instructions, higher in BYI-A, BYI-D and lower in BYI-S [38].

### Statistical analyses

For the statistical analyses, R version 3.5.0 (R Foundation for Statistical Computing, Vienna, Austria) and for descriptives, IBM SPSS Statistics for Windows, version 25.0. (IBM Corp, Armonk, NY) were used. The study group was described by descriptive statistics such as mean (*M*), min and max and standard deviation (SD) for continuous variables and number (*n*) and percentage (%) for categorical. Comparisons between diagnoses and gender (independent groups) were performed by using *t* test, comparisons between parents (dependent groups) by paired *t* test and comparisons with reference groups’ values by using one-sample *t* test. In order to evaluate agreement in ratings between children, adolescents and mothers and fathers, the intraclass correlation coefficient (ICC) was calculated using the ICC function in package psych. ICC coefficients were interpreted according to guidelines, as < 0.40 poor, 0.40–0.59 fair, 0.60–0.74 good and 0.75–1.0 excellent agreement [44]. Multivariable analyses of covariance (ANCOVA) were used to evaluate factors related to the rating scales. Variables included as possible independent predictors were gender, age, gestational age, the presence of anorectal malformation or not, number of procedures in anaesthesia up to 8 years, mother’s BAI score and mother’s BDI score. Correlations between overall HRQoL in DCGM-37 and the results from BYI-A, BYI-D and BYI-S, between parents’ BAI and BDI, and further between parents’ BAI and BDI and their assessments of their children’s HRQoL were estimated by using Pearson correlation and were, according to Cohen, interpreted as strong if *r* ≥ 0.50 [45]. The significance level was set to *p* < 0.05. No adjustment for multiplicity was performed. For comparisons of the scores of the study group, values from reference samples are presented in the results.

### Ethical considerations

Answering questionnaires about psychological well-being might activate psychological distress. For those children/adolescents and parents who scored very elevated or severe anxiety and depression a letter was sent out confirming our findings of their ratings and giving information on available health services to contact if needed.

## Results

The questionnaires were returned by 40 children or adolescents, 38 mothers and 33 fathers. Out of the children and adolescents, 39 were diagnosed with VACTERL association and one child with VACTERL with hydrocephalus (VACTERL-H). Patient characteristics and parents’ reports on complications and physical symptoms during the last year completed for 24 children and adolescents are presented in Table 1.

**Table 1:** Children’s/adolescents’ characteristics, *N* = 40 and reported complications/symptoms during last year, *n* = 24

Characteristics	
Sex, boy/girl, number (*n*)	22/18
Age, years, mean (min–max)	12.8 (8.1–17.7)
GA, full weeks, mean (min–max)^a^	37.0 (30–41)
Malformations, total number in the group, *n* (%)	
Vertebral	28 (70.0)
Anorectal	26 (65.0)
Cardiac	23 (57.5)
Tracheo-oesophageal	24 (60.0)
Renal	18 (45.0)
Limb	11 (27.5)
Anorectal + Tracheo-oesophageal	11 (27.5)
Number of procedures with anaesthesia^b^, mean (min–max)	
Until 1 year of age^a^	3.8 (1–13)
Until 5 years of age^a^	7.8 (1–25)
Until 8 years of age^c^	9.5 (1–28)
Until study^c^	11.3 (2–33)
Reported complications/symptoms last year^d^, *n*	
No complications/symptoms	8
Bowel dysfunction	8
Pain (stomach, back, legs, head)	5
Upper gastrointestinal symptoms (nausea, GE reflux symptoms, vomiting)	4
Urine bladder dysfunction	3
Airway symptoms (cough, asthma)	2
General tiredness or limitations	2
Limb surgery	1

^a^37 values available

^b^Imaging procedures not included

^c^36 values available

^d^Reports of 24 children/adolescents

### Health-related quality of life

**Fig. 1:**
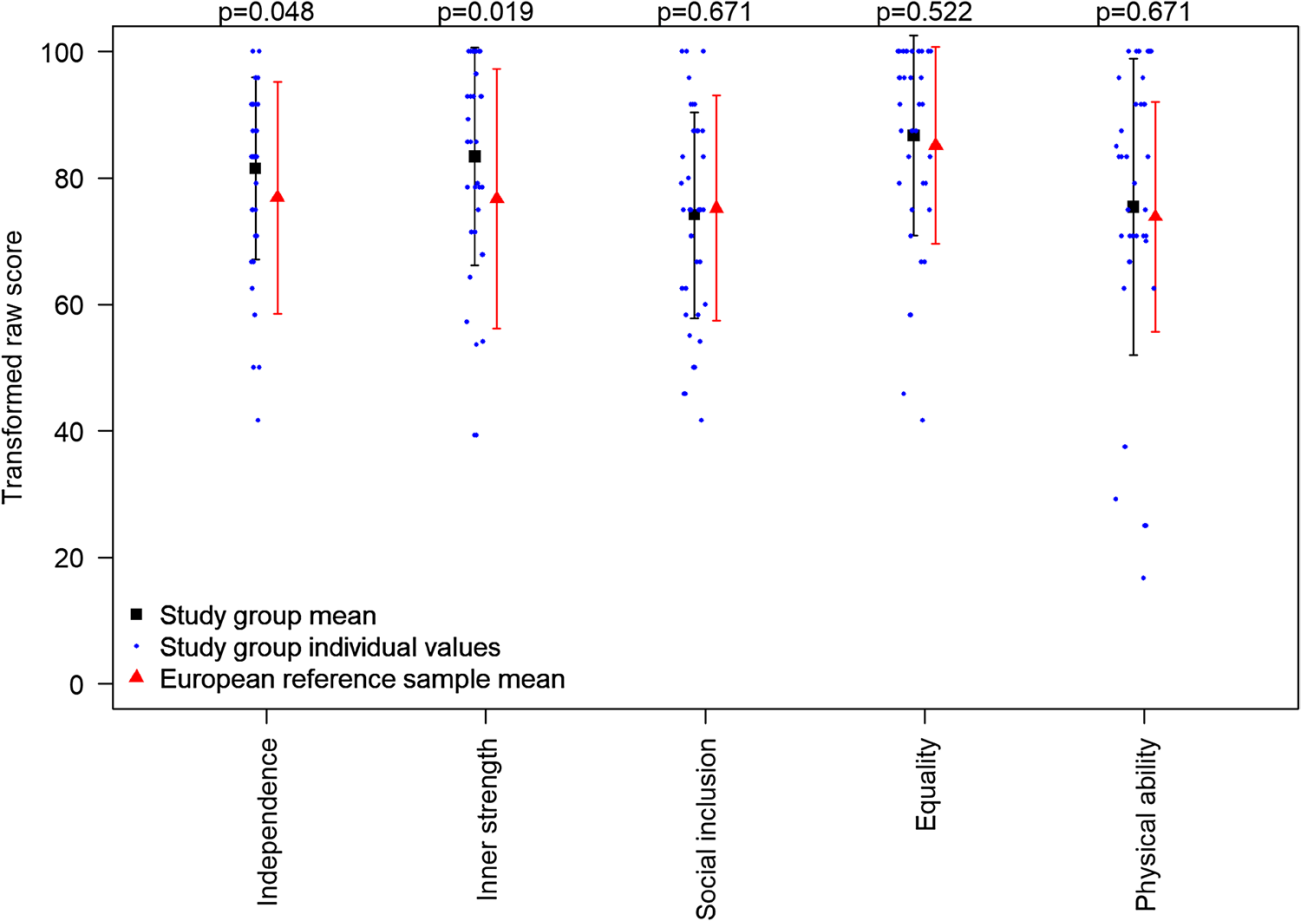
Self-reported HRQoL in DISABKIDS among children and adolescents. Error bars with mean and standard deviation. One-sample *t* test used for comparisons

The overall self-reported HRQoL in children and adolescents with a VACTERL diagnosis was *M* = 80.4, and comparable to children with asthma (*M* = 80.2) and diabetes (*M* = 79.5) in the European reference sample [10]. Significantly higher scores were found in the subscales of independence and inner strengths, compared to the European reference sample with chronic diseases, *p* < 0.05 (Fig. 1). Children and adolescents with VACTERL reported lowest scores in the subscales of social inclusion and physical ability. Results from all subscales are presented in Table 2. When comparing presence or non-presence of a specific malformation, significantly higher scores were reported by children and adolescents with vertebral defects compared to those without, in overall HRQoL and all subscales, *p* < 0.05. Furthermore, children and adolescents with cardiac defects scored significantly lower compared to those without this malformation in social inclusion, mean 69.4 versus 80.5, and equality, mean 82.4 versus 92.6, *p* < 0.05. In the multivariable analysis, significant associations were found between number of procedures in anaesthesia up to 8 years and social inclusion (coefficient − 1.40, 95% CI − 2.53 to − 0.28) and between mothers’ BAI and physical ability (coefficient − 1.72, 95% CI − 3.05 to − 0.38).

**Table 2:** DISABKIDS (DCGM-37) results in study group compared to European reference sample with various chronic conditions (The DISABKIDS Group Europe) [10]

Children’s/adolescents’ reports				
	Study group, boys	Study group, girls	Study group	European
	*n* = 22	*n* = 18	*N* = 40	*N* = 1152
Independence	82.6 (15.0)	80.3 (14.0)	81.6 (14.4)*	76.9 (18.3)
Inner strength	84.6 (19.4)	82.0 (14.6)	83.4 (17.3)*	76.7 (20.6)
Social inclusion	72.0 (16.0)	76.7 (16.8)	74.1 (16.3)	75.2 (17.8)
Equality	86.7 (17.5)	86.8 (14.1)	86.8 (15.9)	85.2 (15.6)
Physical ability	76.4 (22.6)	74.3 (25.1)	75.4 (24.4)	73.8 (18.2)
Overall HRQoL^a^	80.7 (16.3)	80.1 (13.9)	80.4 (15.0)	77.0 (14.2)

Parents’ reports of children’s HRQoL				
	Mothers (M)	Fathers (F)	M + F	European
	*n* = 38^b^	*n* = 31^c^	*N* = 69^d^	*N* = 1061
Independence	74.9 (16.0)	73.9 (16.4)	74.5 (16.1)	76.6 (17.2)
Inner strength	74.1 (17.9)	75.7 (19.5)	74.8 (18.6)	71.6 (20.5)
Social inclusion	70.8 (17.4)	70.3 (16.7)	70.6 (16.9)	74.3 (17.6)
Equality	81.3 (16.7)	80.0 (20.4)	80.7 (18.4)	80.9 (16.8)
Physical ability	70.0 (21.8)	72.6 (18.4)	71.2 (20.3)	70.2 (18.3)
Overall HRQoL	74.8 (15.9)	74.6 (16.6)	74.7 (16.1)	74.9 (14.6)

Transformed raw score 0–100. Mean (SD). Higher values indicate better HRQoL

*Significant difference from European reference sample, p < 0.05. One-sample *t* test

^a^Based on 31 items

^b^n = 37 in equality, overall HRQoL

^c^n = 32 in social inclusion

^d^n = 68 in equality, overall HRQoL, *n* = 70 in social inclusion

The children’s overall HRQoL reported by the parents was *M* = 74.7 and comparable to the parents’ scoring in the European sample of children with various chronic conditions [10]. No significant differences were found between the HRQoL scores of mothers and fathers. In accordance with the children, the parents reported the lowest scores in social inclusion and physical ability (Table 2). Mothers of children with cardiac defects reported their children’s equality significantly lower than mothers of children without this malformation, mean 76.6 versus 87.5, *p* < 0.05.

The intraclass correlation coefficient (ICC) between the children’s, mothers’ and fathers’ assessments for 29 complete triads are presented in Table 3. Agreement in overall HRQoL was excellent between children and mothers and good between children and fathers. In the subscales, fair to excellent agreement was found between children and mothers and fair to good between children and fathers. Agreement between mothers’ and fathers’ assessment of their child’s HRQoL was excellent in overall HRQoL and in all subscales with the exception of inner strength.

**Table 3:** Intraclass correlation coefficient (ICC) between children’s/adolescents’, mothers’ and fathers’ reports in DISABKIDS in 29 families

Subscale	Children–mothers		Children–fathers		Mothers–fathers	
	ICC	95% CI	ICC	95% CI	ICC	95% CI
Independence	0.58	0.27–0.78	0.58	0.28–0.78	0.80	0.62–0.90
Inner strength	0.72	0.48–0.86	0.53	0.20–0.74	0.71	0.47–0.85
Social inclusion	0.66	0.38–0.82	0.53	0.22–0.75	0.92	0.84–0.96
Equality	0.76	0.55–0.88	0.74	0.52–0.87	0.76	0.56–0.88
Physical ability	0.71	0.47–0.85	0.57	0.26–0.77	0.87	0.74–0.94
Overall HRQoL	0.75	0.54–0.88	0.62	0.34–0.80	0.87	0.74–0.94

Interpretation of ICC according to Cicchetti [44]: < 0.40 poor, 0.41–0.59 fair, 0.60–0.74 good, 0.75–1.00 excellent

### Anxiety, depression and self-concept of children and adolescents

**Fig. 2:**
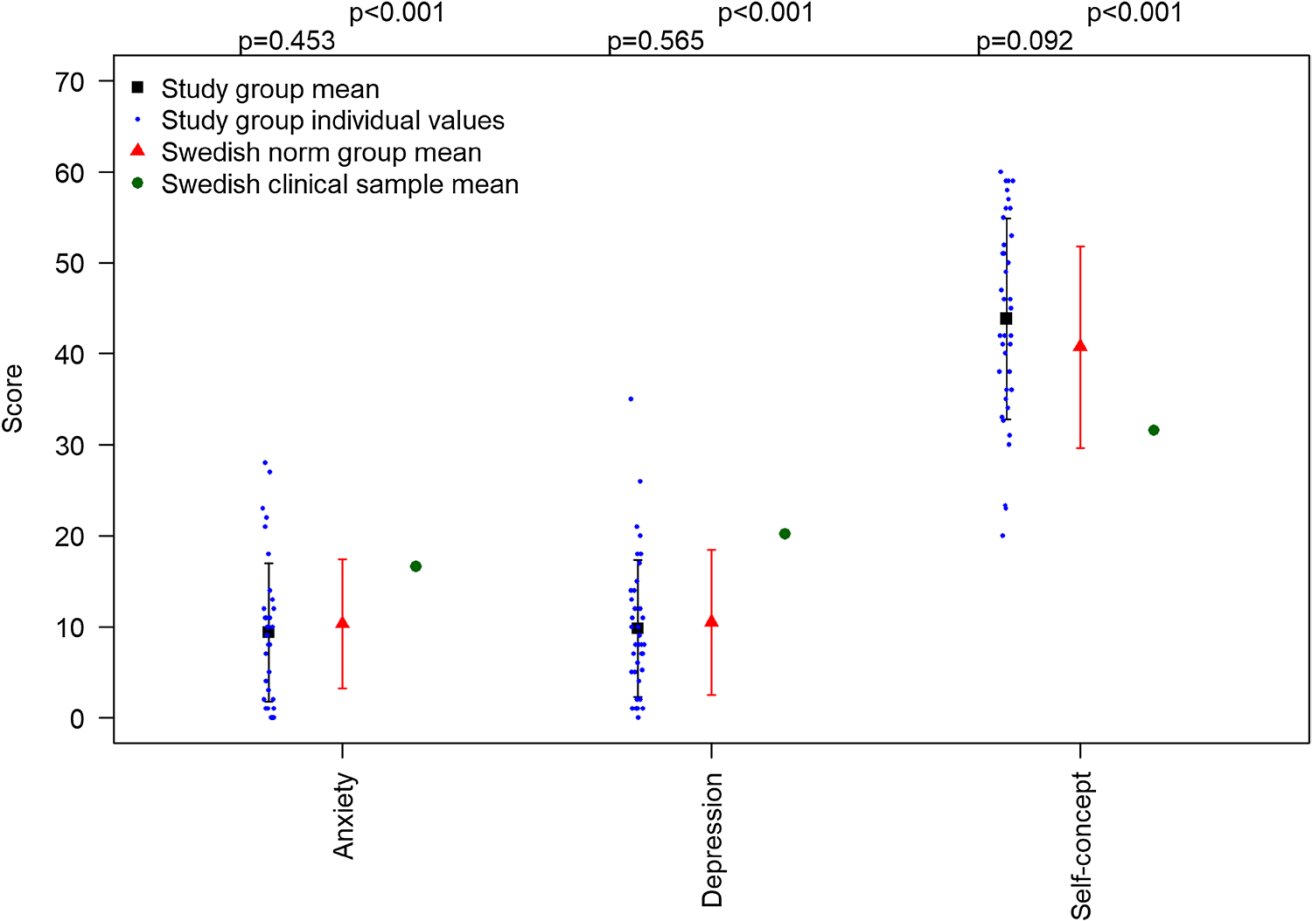
Beck Youth Inventory among children and adolescents. Error bars with mean and standard deviation. One-sample *t* test used for comparisons

Results from the BYI-scales are presented in Table 4. There were no significant differences between the study group and the Swedish norm group. The study group reported significantly lower anxiety, depression and significantly higher self-concept than a Swedish clinical sample (*p* < 0.001) (Fig. 2). According to the BYI guidelines [37], four boys and one girl reported very elevated levels of anxiety and five boys and one girl very elevated levels of depression. Additionally, the scores of one boy and two girls indicated very low self-concept (Table 4). When comparing presence or non-presence of a specific malformation, significantly higher mean scores in self-concept were reported by children and adolescents with vertebral defects compared to those without, mean 47.1 compared to 35.8 (*p* = 0.003). Furthermore, children and adolescents with cardiac defects reported significantly lower scores in self-concept, mean 40.4 compared to 48.2 in those without this malformation, *p* < 0.05. In the multivariable analysis no significant associations were found.

**Table 4:** Mean (SD) scores in the BYI-scales of the children and adolescents in the study group compared to Swedish norm group and a clinical sample (Beck et al. [38])

Group	BYI-A	BYI-D	BYI-S
Study group	9.4***	9.8***	43.9***
Swedish norm group	10.3	10.5	40.8
Swedish clinical sample	16.6	20.2	31.6

	Boys	Girls	Boys	Girls	Boys	Girls
Study group^a^	8.2 (8.9)	10.8 (5.6)	9.1 (9.0)	10.6 (5.4)	46.2 (10.9)	41.2 (10.9)
Swedish norm group^b^	8.6 (6.4)	12.3 (7.2)	8.7 (6.9)	12.5 (8.5)	41.8 (11.0)	39.6 (11.1)

	Classification as per percentiles in study group^c^					
Average	15	15	15	16		
Slightly elevated	2	2	1	1		
Very elevated	4	1	5	1		
High					6	3
Average					12	11
Somewhat low					1	2
Very low					1	2

***Significant difference from Swedish clinical sample, p < 0.001. One-sample *t* test

^a^Study group BYI-A and BYI-D: boys *n* = 21, girls *n* = 18, BYI-S: boys *n* = 20, girls *n* = 18

^b^Swedish norm group aged 9-18, boys *n* = 1246, girls *n* = 1112

^c^Classification as per percentiles [38]

BYI-A & BYI-D: average: < 74th, slightly elevated: 75th–89th, very elevated: ≥ 90th percentile

BYI-S: high: ≥ 90th, average: 26th–89th, somewhat low: 11th–25th, very low: ≤ 10th percentile

A strong positive correlation was found between HRQoL and self-concept in the study group, while strong negative correlations were found between HRQoL and anxiety and depression (Table 5).

**Table 5:** Pearson’s correlation coefficient between self-reported overall HRQoL and anxiety, depression and self-concept in the study group

	Anxiety	Depression	Self-concept
HRQoL	− 0.66	− 0.76	0.74

Classification according to Cohen [45]: *r* ≥ 0.50 strong correlation

### Anxiety and depression in parents

Parents’ reports on the BAI and the BDI are presented in Table 6. Mothers reported significantly higher scores on depression than fathers, mean 9.9 compared to 5.7 (*p* < 0.05), while no significant difference was found in anxiety. According to the suggested classifications [39] five mothers and two fathers reported severe anxiety and four mothers and one father moderate anxiety. Furthermore, four mothers and one father reported severe depression and two mothers and one father moderate depression [40]. Strong correlations were found between self-reported anxiety and depression for mothers as well as for fathers, (Pearson *r* = 0.83 and 0.76, respectively). Scores of self-reported anxiety were significantly higher in mothers of children with limb abnormalities, mean 17.3, compared to mothers of those without this malformation, mean 8.3, *p* < 0.05. No significant differences in mothers’ and fathers’ self-reported depression were found when comparing presence and non-presence of a specific malformation in the children. Correlations between the parents’ scores of BAI and BDI and their assessments of HRQoL of their children were found only between mothers’ BAI and the children’s physical ability (Pearson *r* = − 0.52).

**Table 6:** Scoring of anxiety and depression of mothers (M) and fathers (F) in BAI and BDI-II scales

	BAI			BDI-II		
	Mothers *n* = 34	Fathers *n* = 30	M + F *n* = 64	Mothers *n* = 34	Fathers *n* = 29	M + F *n* = 63
Mean (SD)	10.7 (11.8)	6.4 (9.9)	8.7 (11.1)	9.9 (11.1)*	5.7 (7.0)	8.0 (9.6)

	Level of anxiety^a^			Level of depression^b^		
	Mothers	Fathers	M + F	Mothers	Fathers	M + F
Minimal	21	24	45	25	27	52
Mild	4	3	7	3	0	3
Moderate	4	1	5	2	1	3
Severe	5	2	7	4	1	5

*Significant difference between mothers and fathers, p < 0.05. Paired *t* test

^a^Suggested levels of interpretations of anxiety from Beck and Steer [39]. Minimal: 0–7, mild: 8–15, moderate: 16–25, severe: 26–63

^b^Suggested levels of interpretations of depression from Beck et al. [40]. Minimal: 0–13, mild: 14–19, moderate: 20–28, severe: 29–63

## Discussion

In this Swedish multicentre study including children and adolescents aged 8–17 years with VACTERL association, the HRQoL was basically comparable to the European reference sample with chronic conditions [10], while significantly higher scores were found in the subscales of independence and inner strength [10]. The psychological well-being was similar to the norm group of Swedish school children and more favourable than in a clinical sample. The parents’ self-reports of anxiety and depression were comparable to non-clinical samples.

In the DCGM-37, the children and adolescents in our study group scored higher in the mental domain and similarly in the social and physical domains compared to the European reference sample. The highest scores (*M* = 86.8) were found in the subscale of equality which was originally called social exclusion, where low scores indicate feelings of being stigmatised and left out. Malformations such as limb deficiency [46], cardiac [47] or bowel dysfunction [48] could affect social functioning [49] and psychosocial HRQoL [13]. Hence, high scores suggest that the children and adolescents in our study group to a large extent did not perceive themselves as being excluded due to their health conditions.

The favourable results of the children and adolescents in our study group could be explained by the congenital nature of the health conditions. Children born with a condition may integrate their differences as part of their self-identity resulting in easier adjustment [50] and HRQoL similar to healthy controls [51] and better than in those with an acquired disease [52].

In the parents’ reports on the children’s HRQoL, scores were comparable to those of parents in the European sample of children with various chronic conditions [10]. The ICC values of VACTERL children’s HRQoL showed fair to excellent agreement between children and mothers and fair to good agreement between children and fathers. Note however, that the 95% confidence intervals were quite wide and ranged from poor to excellent. Trends have been reported suggesting that parents of children with health conditions underestimate, while parents of healthy children overestimate their child’s HRQoL [53]. On the other hand, there are previous conflicting reports regarding parents of children with various chronic conditions scoring their child’s HRQoL both lower [36, 54] and higher [55] than the children do themselves [56]. There might be other factors besides the health status of the child, affecting the agreement in reporting of HRQoL between parents and children with chronic conditions. Parent’s own HRQoL [57], distress [57] or worries [58] for the child might influence their perception of the HRQoL of the child. Furthermore, variation has been found in subscales with closer agreement between parents and children in the physical domains compared to emotional and social functioning domains [51, 57].

In the present study, no significant differences were found between mothers’ and fathers’ ratings of the children’s HRQoL, while similar ratings in previous studies have reported differences between parents [59]. Closer agreement has been found between fathers’ and children’s reporting than between mothers’ and children’s, which could be partially explained by cultural factors, overprotection by mothers and fathers’ increased involvement in the child [55]. Conversely, other studies have reported larger differences between fathers’ and children’s scoring than between mothers’ and children’s [57]. The mothers might more often than the fathers take closer care of the child both in their treatment of the health condition and in the daily care, which could contribute to closer proxy reports of mothers [57]. In our small study sample these differences might not be detected. An alternative explanation might be that both parents were actively involved in the treatment and care.

The psychological well-being measured by BYI was comparable to the norm group of Swedish school children with respect to anxiety, depression and self-concept [38] and enhanced compared with the clinical sample [38]. These findings are in line with Athanasakos el al. who did not find increased anxiety or depression compared to controls in children, adolescents and young adults with ARM, in which half of the group had a VACTERL diagnosis [27]. In spite of these results, previous predominantly small studies have reported higher levels of depression in children with CHD than in healthy controls [22] and self-reported depression in approximately one in four children and adolescents with ARM [24, 25]. Therefore it is important to keep the risk of reduced psychological well-being in mind and observe and detect children and adolescents in need of extra support.

Significantly higher scores in DCGM-37 and BYI-S were found in children and adolescents with vertebral defects compared to those without, while significantly lower scores in social inclusion, equality and BYI-S were found in children and adolescents with cardiac defects. The VACTERL condition is, by definition, heterogeneous, and since all study participants had a combination of different malformations, it is not possible to explain why specific malformations would have more or less impact on HRQoL and psychological well-being. The findings may be related to the characteristics of our sample, but as a study of children with VACTERL association, the results are of importance for future investigations. Additionally, ARM is a major malformation which could be expected to influence HRQoL and psychological well-being [13, 24, 25, 49]. Our small study sample may explain that such effects were observed in the multivariable analysis without being significant.

In the multivariable analysis of covariance significant association was found between the number of procedures in anaesthesia up to 8 years and self-reported social inclusion. In line with these findings decreased HRQoL with increasing number of surgeries was reported in children with CHD [17]. An explanation might be that more absence from social contexts such as school and friends [60], may entail a perception of reduced social inclusion. Furthermore, a significant association was found between mothers’ BAI and the children’s and adolescents’ reported physical ability. Hypothetically, these findings could be related to more physical limitations of the child evoking higher anxiety in the mothers. On the contrary, Skreden et al. [30] did not find any association between the child’s health and daily function and state anxiety and psychological distress in parents of children born with malformations [30]. Lower employment—financial—and educational status may instead be stronger predictors of the parents’ negative psychological outcomes [30, 31].

The parents’ reported scores of anxiety in our study were comparable to Finnish [41], US [42] and Spanish [43] non-clinical samples and regarding depression the scores were comparable to a non-clinical Norwegian sample [40]. Even though we did not find any significant differences in the results of BAI between mothers and fathers, nine out of 34 (26%) mothers’ scores were classified as moderate or severe anxiety and 3/30 (10%) among fathers. Corresponding classifications for depression were 6/34 (18%) of scorings of mothers and 2/29 (7%) of fathers. Among parents of children with chronic health conditions, 16% reported fulfilled criteria for anxiety disorder [61] and 21% for depressive disorder [62]. When comparing parents of children with congenital anomalies with parents of healthy children, levels similar or exceeding norms of psychiatric outpatients were found of anxiety in 15% versus 7% and of depression in 18% versus 10% [31]. Similarly, clinically important psychological distress has been reported in 30% of parents of children with congenital anomalies versus 21% in parents of healthy children, clinically important state anxiety in 27% versus 14% [30], and moderate or severe depressive symptoms in 27% versus 14%, respectively [33]. Thus overall, parents of children with health conditions have reported higher levels of psychological symptoms than parents of healthy children. Furthermore, when comparing reports of mothers and reports of fathers of children with congenital anomalies, levels similar or exceeding norms of psychiatric outpatients have been reported of anxiety in 18% versus 11% and of depression in 21% versus 10% [31]. Similarly, clinically important psychological distress has been found in 36% of mothers versus 23% of fathers of children with congenital anomalies [30]. Also, more depressive symptoms have been demonstrated in mothers compared to fathers [33]. Moreover, in general Swedish samples, higher levels of depression and anxiety have been reported in women compared with men [63, 64]. The responsibility for care of children with a chronic condition may not be divided equally between the parents [31, 65] with mothers taking greater responsibility for the medical and daily care of the child, which may partially explain the results. Since some parents of children with complex health conditions might suffer from increased anxiety and depression it is important that those in need of support are detected by healthcare providers.

## Methodological considerations

### Strengths

To our knowledge this is the first study to investigate HRQoL and psychological well-being in children and adolescents with VACTERL association. The findings increase the understanding of the HRQoL and psychological well-being in children and adolescents with VACTERL association and also of the psychological well-being in their parents. The study is strengthened by the multicentre design including patients from three out of four paediatric surgical centres in Sweden, which enables the evaluation of a fairly large sample size considering the low birth prevalence of VACTERL association.

### Limitations

Generalisability of the study results is limited by the response rate of around 60%, which entails a risk of selection bias. Dropout analysis was not possible due to the lack of ethical approval. Furthermore, the choice of HRQoL instruments must be considered in the interpretation of results, since generic and diagnosis-specific instruments might reveal different outcomes. No adjustment for multiplicity was performed and all *p* values should therefore be interpreted with caution. Moreover, the children with VACTERL association form a heterogeneous group with various types and severities of malformations which might contribute to large variations in the results and could also impede definite conclusions. Finally, the relatively small group of respondents from a comparatively homogenous context may limit generalisability to other regions.

## Conclusions

Although children and adolescents with VACTERL association reported HRQoL similar to those of European children with other chronic conditions, their psychological well-being was comparable to that of Swedish school children in general and significantly higher than a clinical sample. The parents’ self-reports of anxiety and depression were comparable to non-clinical samples. Nevertheless, since there were some individuals among both children and parents in need of attention and extra support the results call upon awareness to observe and detect reduced psychological well-being. The knowledge obtained from the study is valuable when counselling parents on the prognosis of children with VACTERL association.
